# 

**Published:** 1894-08

**Authors:** 


### CONTENTS.

### COMMUNICATIONS.

Restoratives and Resuscitants .......... 365
Hypnotism .......... 380
Anæsthetizing Sensitive Dentine .......... 383
Electricity and "Painless Dentistry" .......... 394
Persistent Vitality .......... 399
An Act for the Regulation of the Practice of Dentistry in the State of Michigan .......... 403

### SELECTIONS.

Indications for Cocaine in Surgical Operations .......... 408
Successful Removal of a Denture from the Œsophagus .......... 409
A Curious Case .......... 409
Obtundents for Sensitive Dentine .......... 410
Glycerine .......... 410
Amyl Nitrite .......... 410
What Caused Death .......... 410

### COMMENCEMENTS.

University of Minnesota—Dental Department .......... 411
Chicago College of Dental Surgery .......... 412
Northwestern University Dental School .......... 412
University of Buffalo—Dental Department .......... 413
University of California—College of Dentistry .......... 413
Harvard University—Dental Department .......... 414

### NOTICES.

The American Dental Society of Europe .......... 414
Alumni Reunion .......... 415
Connecticut State Dental Association .......... 416

### OBITUARY.

Dr. R. B. Winder .......... 416

